# Preparedness level of frontline healthcare professionals in Tshwane regarding the COVID-19 pandemic

**DOI:** 10.4102/safp.v64i1.5341

**Published:** 2022-01-24

**Authors:** Tombo Bongongo, Indiran Govender, Doudou K. Nzaumvila, Olga M. Maphasha

**Affiliations:** 1 Department of Family Medicine and Primary Health Care, School of Medicine, Sefako Makgatho Health Sciences University, Pretoria, South Africa; 2 Department of Family Medicine & Primary Health Care, School of Medicine, University of Pretoria, Pretoria, South Africa

**Keywords:** preparedness level, frontline healthcare professionals, Tshwane, coronavirus 2019 pandemic

## Abstract

**Background:**

Facing the highly transmissible viral infection referred to as the coronavirus disease 2019 (COVID-19) that threatens human life, South African frontline healthcare professionals have faced a major challenge. Being one of the African countries with a higher prevalence of COVID-19 cases, this study aimed to assess the preparedness levels of emergency doctors, nurses and clinical associates in the Tshwane district of South Africa regarding the COVID-19 pandemic.

**Methods:**

This was a cross-sectional survey using a developed and piloted online questionnaire. It was conducted in the casualty departments (emergency medical units) of seven hospitals in the Tshwane district of South Africa. Only emergency doctors, nurses and clinical associates took part.

**Results:**

Of the 87 respondents, the mean age was 33.9 years and the minimum and maximum ages were 22 and 62 years, respectively; 37 (42.5%) were aged 26–30 years, 54 (62.1%) were females, 46 (52.9%) were single persons, 35 (40.2%) were medical officers, 42 (48.3%) were healthcare professionals with 0–5 years of experience and 21 (24.1%) were from a provincial tertiary hospital. Of the healthcare professionals, 63 (72.4%) were assessed as being prepared regarding the COVID-19 pandemic.

**Conclusion:**

The current online survey demonstrated a good preparedness level regarding the COVID-19 pandemic management amongst frontline healthcare professionals working in seven hospitals of the Tshwane district. An educational training programme on disaster management or the COVID-19 pandemic should be implemented to ensure that all frontline healthcare professionals are adequately prepared for current and future outbreaks.

## Introduction

Preparedness, as defined by the United Nations International Strategy for Disaster Reduction (UNSDR), consists of knowledge and capabilities established by an individual or an organisation to efficiently anticipate or respond to a forthcoming or existing threat or circumstances. The preparedness can be high or low or good or bad.^1^

By working long hours and at times in a stressful environment, healthcare workers are continuously exposed to different threats, which can lead to them becoming sick. This hard work was recognised and honoured by the World Health Organization (WHO) through the declaration of the Decade of Human Resources for Health.^2^

In connection with what has been recognised by the WHO, female healthcare professionals (HCPs) in Lahore, Pakistan, felt the weight of the coronavirus disease 2019 (COVID-19) pandemic, and many developed anxiety and stress because of an unwelcoming working environment and family pressure. This pressure might be raised because of a lack of training as well as non-existence of incentives whilst working during such difficult times. The absence of a proper surveillance system in the healthcare system of the country also contributed to the increased psychological pressure described amongst female HCPs.^3^

Although the number of HCPs matters when looking at how to tackle a health disaster, more often professionals in health are unwilling to work during times of disaster.^4^ Certain strategies may assist in enhancing employees’ willingness to take part in a response to an outbreak, such as promoting pre-event plans and ensuring adequate supplies of personal protective equipment (PPE), availability of vaccines and awareness and preparedness through education.^5^

A UK systematic review, looking at the literature focusing on the willingness of HCPs to work during a disaster, shows that there are variables that have to be considered whilst facing difficulties. Concerns for family as well as personal safety play a large role in the HCPs’ willingness to work during a time of disaster. Lack of PPE can be a hindrance to the preparedness of HCPs to work during difficult times.^6^

In Yemen, a cross-sectional study with the preparedness of healthcare workers as one of the objectives concluded that they had good preparedness regarding the management of COVID-19 infection overall, with male participants scoring higher than female participants. The results of this study encouraged the healthcare authority and different stakeholders to attend to the alleged barriers relating to the preparedness of the healthcare workers to prevent and control infection in the country.^7^

With COVID-19 devastating the world, a study targeting the knowledge and preparedness levels of physicians and nurses regarding the pandemic was conducted in Libya in 2020. In terms of participation, two-thirds of the sample was constituted by physicians and one-third by nurses. The study showed that almost equal numbers of both groups received training on how to use PPE, but less than one-third of each group assessed themselves as prepared in terms of managing COVID-19 patients. This reflected a low preparedness level amongst frontline workers for handling COVID-19 clients, and the need for a programme of education and training for healthcare workers to ensure a good preparedness level regarding the COVID-19 pandemic was emphasised.^8^

Using a multicentre for disease control checklist for healthcare personnel’s preparedness for the transport and arrival of confirmed as well as possible COVID-19 cases, an audit was conducted in various healthcare centres amongst clinicians (junior doctors and nurses) and non-clinicians in Pakistan. The outcome of this audit showed a discrepancy in the results: clinicians scored higher on knowledge than non-clinicians. After educating the non-clinicians, the results of a second audit showed an improvement amongst this category.^9^ There is therefore a need to educate non-clinicians, because this can reduce the transmission rate and the non-clinicians are sharing the same setting with the clinicians and the patients.

Despite the public health interventions noted in a few African countries, including South Africa, no comprehensive assessment of sub-Saharan Africa’s preparedness was performed at the beginning of the emergence of the highly transmissible viral infection referred to as COVID-19.^10^ Considering the high transmission rate and the morbidity as well as mortality of this viral infection, on the one hand, and the vocational training of HCPs that binds them to the work of saving lives on the other, this survey aims to assess the preparedness level of frontline HCPs in Tshwane district of South Africa regarding the COVID-19 pandemic.

## Methods

### Study design

This was a cross-sectional survey using an adapted online questionnaire. The survey was conducted in the casualty departments or emergency medical units of seven hospitals in the Tshwane district of South Africa. The doctors, nurses and clinical associates working in the emergency department participate in the current study.

### Sample size and sampling

All medical doctors (specialists and medical officers), all nurses and all clinical associates working in the casualty or emergency medical departments of seven hospitals located in the Tshwane district were targeted. Convenience sampling was applied, in that only the completed online questionnaires recorded on the Excel spreadsheet of the researcher, which was regarded as available, were considered.

### Data collection

Data collection took place from November 2020 to April 2021. With the assistance of the information technology personnel of the Department of Family Medicine at the University, an Microsoft Excel spreadsheet was made available in the Google Drive of the researcher. The researcher met physically with all heads of emergency units and presented the survey to them with its aim, objectives and the online questionnaire.

This online questionnaire was adapted from a study that looked at the levels of preparedness and awareness regarding COVID-19 infection in low-resource settings, performed in Libya^8^ and published in 2020. The study assessed the healthcare workers’ levels of preparedness and awareness regarding COVID-19 infection in low-resource settings.^8^ The adapted questionnaire was adjusted in order to respond to the objectives of the Tshwane survey. The final online questionnaire was assessed for test–retest reliability and was piloted in a sample that was not included in the present survey. Frontline HCPs who received the online questionnaire, consented to take part in the survey, went through it and ticked the answers had their answers captured on the Excel spreadsheet that was in the Google Drive of the researcher.

### Data analysis

From the Excel spreadsheet, data were imported to Statistical Package for the Social Sciences (SPSS) software version 26 where descriptive analysis was performed. Continuous variables were summarised by mean, standard deviation, median, interquartile range, minimum and maximum values. Categorical variables were summarised by frequency counts and percentage calculations.

Frontline HCPs who have had training on disaster or COVID-19 management, who were willing to be trained on disaster or COVID-19 management and who can work whilst having PPE and had a positive self-assessment for managing COVID-19 patients were considered as ‘prepared’. The preparedness level was assessed as ‘good’ when the sum of the trainings (done or to be done), willingness to work when there is PPE and a positive self-assessment in managing COVID-19 patients was equal to or above 50%.

In this present survey, the preparedness level is the knowledge and capabilities acquired by an individual (frontline HCP) or organisation (casualty unit) to be able to respond competently to a threat (COVID-19 pandemic).^1^ Considering Table 2 on preparedness, all of the ‘Yes’ as well as the ‘High’ responses supported the preparedness of the HCPs, whilst ‘Not sure’, ‘No’ and ‘Low’ did not. The sum of all the ‘Yes’ answers characterised a good level of preparedness if it was equal to or above 50%.

## Results

With a mean age of 33.9 years and the minimum and maximum ages of 22 and 62 years, respectively, most of the respondents were in the age group of 26–30 years (37; 42.5%), were female (54; 62.1%) and were single (46; 52.9%). Medical officers numbered 35 (40.2%), with 42 (48.3%) HCPs having 0–5 years of practice. Twenty-one (24.1%) were from a Provincial Tertiary Hospital (Table 1).

**TABLE 1:** Baseline characteristics.

Age group (years)	Frequency	Percentage
21–25	11	12.6
26–30	37	42.5
31–35	12	13.8
36–40	3	3.4
41–45	12	13.8
46–50	7	8.0
51–55	3	3.4
56–60	12	13.8
61–65	2	2.3
Total	87	100.0
**Gender**		
Female	54	62.1
Male	33	37.9
Total	87	100.0
**Marital status**		
Divorced	1	1.1
Married	37	42.5
Single	46	52.9
Cohabiting	1	1.1
Widowed	2	2.3
Total	87	100.0
**Level of education**		
Clinical associate	14	16.1
Enrolled nurse	14	16.1
Medical officer	35	40.2
Medical specialist	8	9.2
Professional nurse	16	18.4
Total	87	100.0
**Years of experience**		
0–5	42	48.3
6–10	17	19.5
11–15	12	13.8
16–20	6	6.9
21+	10	11.5
Total	87	100.0
**Hospital**		
Dr George Mukhari Academic	12	13.8
Kalafong	21	24.1
Mamelodi	10	11.5
Odi	12	13.8
Pretoria West	7	8.0
Steve Biko cademic	9	10.3
Tshwane District	16	18.4
Total	87	100.0

In terms of preparedness levels, 32 (36%) respondents were trained in health disaster management, whilst 27 (31%) had previous experience in health disaster management, and 38 (43.7%) had formal training in COVID-19 pandemic management. Many trusted in the hospital safety plan (*n* = 50; 57.3%) as well as in their trained colleagues’ preparedness in terms of COVID-19 management (*n* = 60; 69.0%). The majority of respondents were willing to participate in health disaster management (*n* = 77; 88.5%). Most rated their self-efficacy in COVID-19 management as high (*n* = 62; 71.3%) and most agreed that they could manage a COVID-19 patient if they had full PPE (*n* = 80; 92.0%) (Table 2).

**TABLE 2:** Preparedness levels amongst the respondents.

Elements of preparedness	Frequency	Percentage
**I had a training in health disaster management**		
No	49	56.3
Not sure	6	6.9
Yes	32	36.0
Total	87	100.0
**I have previous experience in health disaster management**		
No	59	67.8
Not sure	1	1.1
Yes	27	31.0
Total	87	100.0
**I had formal training in COVID-19 pandemic management**		
No	44	50.6
Not sure	5	5.7
Yes	38	43.7
Total	87	100.0
**I put my trust in the hospital safety when it comes to COVID-19 pandemic management**		
No	24	27.6
Not sure	13	14.9
Yes	50	57.5
Total	87	100.0
**I put my trust in my colleagues’ preparedness to react to the COVID-19 pandemic (trained colleagues)**		
No	11	12.6
Not sure	16	18.4
Yes	60	69.0
Total	87	100.0
**I am willing to participate in health disaster management**		
No	8	9.2
Not sure	2	2.3
Yes	77	88.5
Total	87	100.0
**How I rate my self-efficacy in COVID-19 management**		
Low	14	16.1
Not sure	11	12.6
High	62	71.3
Total	87	100.0
**With full PPE, I can manage a COVID-19 patient**		
No	4	4.6
Not sure	3	3.4
Yes	80	92.0
Total	87	100.0

PPE, personal protective equipment; COVID-19, coronavirus disease 2019.

High participation was noted amongst women, and most of them were prepared regarding the COVID-19 pandemic (*n* = 36; 41.4%); men had a low participation, but most were also prepared (*n* = 27; 31.0%). In terms of marital status, there was high participation amongst those who were single, and the majority of them were prepared (*n* = 34; 39.0%). Regarding the level of education, high participation was seen in the category of medical officers, and they also scored high in terms of preparedness level (*n* = 24; 27.6%). Most participants had 0–5 years of experience, with a high preparedness level regarding the COVID-19 pandemic (*n* = 27; 31.0%).

Different socio-demographics were classified in two groups: prepared and not prepared. The outcome has been formulated in frequencies and percentages, as presented in Table 3.

**TABLE 3:** Row percentages comparing the bivariate (prepared vs. non-prepared) variables.

Variable	Prepared		Not prepared		Total	
	Frequency	Percentage	Frequency	Percentage	*n*	%
**Gender**						
Female	36	41.4	18	20.7	54	62.1
Male	27	31.0	6	6.9	33	37.9
Total	63	72.4	24	27.6	87	100.0
**Marital status**						
Divorced	1	1.2	0	0.0	1	1.2
Married	27	31.0	10	11.5	37	42.5
Single	34	39.0	12	13.8	46	52.8
Cohabiting	1	1.2	0	0.0	1	1.2
Widowed	0	0.0	2	2.3	2	2.3
Total	63	72.4	24	27.6	87	100.0
**Level of education**						
Clinical associate	10	11.5	4	4.6	14	16.1
Enrolled nurse	12	13.8	2	2.3	14	16.1
Medical officer	24	27.6	11	12.6	35	40.2
Medical specialist	6	6.9	2	2.3	8	9.2
Professional nurse	11	12.6	5	5.7	16	18.4
Total	63	72.4	24	27.6	87	100.0
**Years of experience**						
0–5	27	31.0	15	17.2	42	48.3
11–15	10	11.5	2	2.3	12	13.8
16–20	5	5.7	1	1.1	6	6.9
21+	7	8.0	3	3.4	10	11.5
6–10	14	16.1	3	3.4	17	19.5
Total	63	72.4	24	27.6	87	100.0

Most frontline HCPs were assessed as ‘Prepared’ (63; 72.4%), whilst 24 (27.6%) were ‘Not prepared’, as indicated in Table 4.

**TABLE 4:** Proportions of respondents who were ‘prepared’ and ‘not prepared’.

Variable	Frequency	Percentage	Total	
			*n*	%
Prepared	63	72.4	63	72.4
Not prepared	24	27.6	24	27.6

**Total**	**87**	**100.0**	**87**	**100.0**

## Discussion

The main objective of this survey was to assess the preparedness level of the frontline HCPs regarding the management of COVID-19 pandemic, and the findings amongst the Tshwane sample demonstrated a good level of preparedness (Table 4). This is consistent with what was found in Yemen.^5^ However, in Yemen, although the overall preparedness was good, male respondents scored higher than female respondents, whilst the opposite was noted from the Tshwane survey. This might be attributed to the high proportion of female participation that characterised the South African sample, as presented in Table 1.

The good preparedness level observed amongst the Tshwane frontline HCPs is also consistent with what was displayed by the clinicians in Pakistan.^9^ Even though there was no comparison between the clinicians and the non-clinicians in Tshwane, the Pakistan study has stretched the need of taking on board all categories of individuals sharing the same hospital environment with clinicians during an outbreak; as all of them are exposed to the same health hazard. Educational training programme as well as the precautionary measures such as use of PPE should not be limited to one category of workers.

Lack of PPE, as mentioned in some studies,^5,6^ was a hindrance to the preparedness of HCPs to work during difficult times, as supported by the high number (92%) of respondents from the Tshwane survey who responded that ‘Yes’ they could manage COVID-19 patients if PPE was available (Table 2). Therefore, health authority should prevent any lack of PPE.

In Tshwane, South Africa, where most of the respondents did not receive formal training on COVID-19 management (Table 2), the preparedness regarding the pandemic was assessed as good; this was contrary to the situation in Libya,^8^ where respondents were trained on the use of PPE but both physicians and nurses had low preparedness. Such Libyan outcome should not constitute a reason for discouraging training, whilst the pandemic is still devastating the communities.

Education and PPE formed part of strategies that enhanced the willingness to take part in the response to an outbreak as demonstrated in the Tshwane survey as well as in various studies.^5,6,8,9^ This implies that the healthcare system has to guarantee good preparedness by ensuring that there is PPE and training the personnel for any future pandemic or every COVID wave that can arise.

### Strengths and limitations

This online survey was conducted during the most critical time of the COVID-19 second wave (November 2020 – April 2021), but the difficulties encountered did not stop the frontline HCPs from expressing themselves through the survey. As the survey did not cover all of the frontline HCPs in the Tshwane health district, the results cannot be generalised to the entire district.

## Conclusion

The present survey demonstrated a good preparedness level amongst frontline HCPs working in seven hospitals of the Tshwane district regarding the COVID-19 pandemic. An educational training programme on disaster management or the COVID-19 pandemic has to be implemented to ensure that all frontline HCPs are adequately prepared for the management of current and future outbreaks.
